# Acceptability of Self-Help Plus and Problem Management Plus interventions among clients and implementation insights from staff of Opioid Agonist Treatment programs in Ukraine

**DOI:** 10.1371/journal.pmen.0000470

**Published:** 2026-06-22

**Authors:** Vitalii Klymchuk, Viktoriia Gorbunova, Vladyslav Romanchuk, Iryna Ivanchuk

**Affiliations:** 1 Department of Social and Applied Psychology, Zhytomyr Ivan Franko State University, Zhytomyr, Ukraine; 2 University of Luxembourg, Esch-sur-Alzette, Luxembourg; 3 State Institution “Public Health Center of the Ministry of Health of Ukraine”, Kyiv, Ukraine; PLOS: Public Library of Science, UNITED KINGDOM OF GREAT BRITAIN AND NORTHERN IRELAND

## Abstract

Self-Help Plus (SH+) and Problem Management Plus (PM+) are two scalable psychological interventions developed by the World Health Organization to reduce stress, improve emotional regulation, and strengthen coping skills among adults experiencing adversity. SH+ is a low‑intensity, group‑based stress‑management course, while PM+ is a brief, individual intervention focused on problem‑solving and behavioural activation. This study explores the acceptability of SH+ and PM+, among staff providing psychosocial support and clients of the opioid agonist treatment (OAT) programs in Ukraine during wartime. A qualitative study was conducted using semi-structured interviews with 12 clients, alongside an analysis of meeting notes from monthly staff management discussions. Data were collected from April to October 2023 (during the second year of the full-scale Russian invasion of Ukraine) at OAT centers in Lviv, Sumy, and Vinnytsia. The SH+ group format created a supportive atmosphere, which contributed to the outcomes despite initial challenges with client engagement. Participants noted that even passive attendance in program sessions was associated with positive impacts, reflecting the intervention’s suitability for clients who struggle with active participation. The key advantage of PM+ was its flexibility in scheduling, which was beneficial given many clients’ unpredictable and chaotic life situations. The personalized nature of PM+ facilitated open dialogue and a deeper exploration of clients’ needs. Clients responded positively to the individual format of PM+, finding it familiar and comfortable. They appreciated the opportunity to be heard in a relaxed and confidential setting. Overall, the study indicates that SH+ and PM+ are acceptable, feasible, and valued by both clients and staff, supporting their potential integration into routine OAT services to help address unmet mental health needs during wartime.

## Introduction

Since 2014, Ukraine has faced a severe crisis due to the ongoing war initiated by Russia, which escalated into a full-scale invasion in 2022. Despite all the efforts to improve the delivery of mental health and psychosocial support, a significant disparity between the growing demand for psychological support and the capacity of the healthcare system to meet these needs [1,2]. People living with opioid use disorder (OUD) are among the most affected, facing additional social and physical challenges, such as interrupted access to OAT medication, displacement, and job-related issues, along with a higher prevalence of depression, anxiety, PTSD symptoms, and suicidal ideation [3].

Support for individuals with OUD in Ukraine is provided through a network of publicly funded healthcare services managed by the National Health Service of Ukraine. These services include Opioid-Assisted Treatment, which is provided at 205 state-funded OAT centers, assisting approximately 17,210 clients [4,5]. Originally, these centers were created to provide treatment using methadone, buprenorphine, and, more recently, buvidal. Over time, OAT centers have transformed into comprehensive support hubs that address the diverse needs of their clients, integrating harm reduction services with psychological, medical, social, and legal assistance, both independently and in collaboration with local NGOs [6–9].

Despite these efforts, integrating specialized psychiatric care into existing healthcare services, including OAT centers, is unlikely to bridge the treatment gap due to workforce shortages, high demand, and financial constraints. It is possible to address these challenges by implementing task-shifting and task-sharing strategies, particularly using scalable psychosocial interventions like Self-Help Plus (SH+) and Problem Management Plus (PM+) [10–14]. Both interventions were designed specifically for populations experiencing adversity and limited access to specialist mental health care, making them suitable for integration into OAT programs in Ukraine and for addressing both general psychological distress and war‑related stressors.

SH+ is a low-intensity, group-based stress-management intervention. It uses an illustrated self-help manual and audio recordings to teach mindfulness, grounding, and cognitive coping strategies. Its goal is to reduce psychological distress and strengthen emotional regulation and resilience among adults exposed to adversity.

PM+ is a brief, individual intervention that combines problem-solving, behavioural activation, stress-management, and social support enhancement. The objective of PM+ is to reduce symptoms of anxiety, depression, and stress by equipping participants with practical skills to cope with daily challenges.

Both interventions were developed by WHO, and designed to be delivered not just by mental health professionals, but also by trained non-specialists [10–14].

Despite intensive studies, their impact on individuals with co-occurring Opioid Use Disorders and other mental health conditions has not been widely explored yet. The recent pragmatic trial, assessing the effectiveness of these interventions, focused on adults with OUD and was conducted in Ukraine, demonstrating promising improvements in mental health outcomes. The SH+ showed additional benefits, a decrease in missed medication doses, and a reduction in PTSD and depression symptoms [15].

The aim of this qualitative part of the study, presented in this paper, was to explore the acceptability of two WHO scalable psychological interventions (SH+ and PM+) among clients and staff of OAT centers in Ukraine during wartime. Specifically, the study sought (1) to assess how acceptable the interventions were in terms of participants’ engagement, perceived usefulness, and willingness to recommend them, and (2) to examine how individual experiences and contextual factors shaped these perceptions within the unique environment of OAT service delivery during an ongoing armed conflict, and (3) to provide recommendations for scaling up the interventions.

## Materials and methods

### Qualitative approach and research paradigm

To study the acceptability of WHO scalable psychological interventions (SH+ and PM+) in OAT programs in Ukraine, a phenomenological approach within an interpretive paradigm was chosen [16]. This qualitative research methodology aligns with the main research idea: to understand the perception of the interventions through the lens of acceptability, and to examine the impact of contextual factors and individual experiences on this perception.

Phenomenology focuses on understanding how individuals make sense of their experiences and the meanings they assign to those experiences, making it well-suited for examining perceptions of acceptability in a complex, emotionally charged context [16]. The interpretive paradigm assumes that reality is socially constructed and that knowledge emerges through interaction between participants and researchers [17]. In this study, it guided our attention to the subjective, context-dependent nature of client and staff experiences during wartime.

This study report follows the Standards for Reporting Qualitative Research (SRQR guideline) [18].

### Researchers’ characteristics and reflexivity

Given the ongoing war in Ukraine, the study was conducted in a context of extreme uncertainty, increased mental health distress, and resource limitations. Acknowledging our roles and potential biases is crucial for understanding the methodological choices made and their impact on the findings. The research team consisted of experts in psychology, mental health, and public health with experience in mental health research, particularly in conflict-affected and resource-limited settings. Our combined expertise shaped the study’s focus on scalable interventions to address mental health gaps in Ukraine’s healthcare system.

#### Assumptions and preconceptions.

We recognized the urgent need for mental health support among individuals with opioid use disorder in Ukraine. We also acknowledged several assumptions that could have influenced our approach: a) the expectation that SH+ and PM+ would be well-received due to their previous success in other humanitarian settings; b) the belief that stigma surrounding opioid use and mental health might pose a barrier to participation; c) the assumption that group-based interventions (SH+) could be more difficult to implement than individual interventions (PM+) due the personal nature of trauma and distress. To mitigate potential biases, we designed the study to allow participants to openly express both positive and negative experiences with the interventions. This ensured that acceptability was assessed from their perspectives rather than our expectations.

#### Positionality and power dynamics.

Conducting research in conflict-affected environments requires careful consideration of power dynamics. As researchers associated with European and Ukrainian institutions, our institutional authority and perceived expertise affected our interaction with participants in OAT centers. To reduce power imbalances: a) we conducted the study in close partnership with OAT center staff, ensuring that research tools were developed with input from those directly working with clients; b) data collection methods (semi-structured interviews and meeting notes) were designed to empower participants, enabling them to share their perspectives freely; c) we emphasized confidentiality and voluntary participation to create a safe environment for open conversation. Despite these efforts, we recognize that our roles as external researchers may have influenced participants’ responses, particularly regarding their readiness to criticize the interventions.

#### Interaction with participants.

Our interactions with OAT staff and clients significantly shaped the research process. As a team with expertise in mental health and public health, we knew our involvement could impact participants’ engagement with the study. Key reflections include the necessity of building trust, as participants may have been initially hesitant to share their experiences due to the sensitive nature of opioid use and mental health. Some individuals might have perceived the research team as associated with healthcare providers or policymakers, which could have affected how they framed their responses. Conducting research with individuals experiencing trauma and mental distress requires a sensitive approach. Researchers ensured that interviews were carried out empathetically, without pressuring participants to share distressing experiences.

#### Methodological choices and reflexivity.

The choice of a qualitative research design, which combines semi-structured interviews with participants and the analysis of staff meeting notes, was influenced by the researchers’ backgrounds in mental and public health. This approach allowed for a nuanced exploration of intervention acceptability, but we recognize certain limitations. Specifically, the thematic analysis was conducted by researchers with prior knowledge of SH+ and PM+, which may have influenced how themes were interpreted. The selection of interview questions was informed by previous research on mental health interventions, which could have shaped the types of responses elicited. The small sample size (12 participants) was a result of feasibility constraints, along with our understanding that individuals with OUD might be reluctant to engage in longer research participation. To mitigate these potential biases, the research team regularly discussed data interpretation, ensuring that emerging themes were grounded in participant narratives rather than in researcher expectations.

### Context

This qualitative study was conducted as part of a pragmatic trial assessing the effectiveness of the SH+ and PM+ interventions for individuals with OUD receiving treatment at OAT centers in Ukraine during the ongoing Russian invasion [15]. While quantitative data on effectiveness is important, the acceptability of the interventions among both clients and staff significantly impacts the potential for broader implementation and sustainable integration of the interventions into other OAT centers in Ukraine.

#### Interventions.

Two WHO-developed structured interventions, Self-Help Plus (SH+) [11] and Problem Management Plus (PM+) [12] were integrated into the existing OAT centers’ services at three pilot sites in Ukraine (Vinnytsia, Lviv, and Sumy) within the frame of a pragmatic trial. The SH+ and PM+ interventions have distinct features tailored to different needs. SH+ is a group-based, low-intensity stress management program based on WHO’s SH+ manual. It incorporates mindfulness and stress management techniques along with audio-visual tools to enhance delivery. In contrast, PM+ is an individual-focused intervention designed to address psychological distress and improve problem-solving skills. It includes behavioral activation, relaxation techniques, and problem-solving strategies combined with emotional regulation.

Their intended use in the Ukrainian context was twofold: (1) to support the mental health of individuals with opioid use disorder, who commonly experience elevated levels of depression, anxiety, and psychosocial instability; and (2) to provide practical coping strategies for managing the additional stressors associated with wartime conditions, such as insecurity, displacement, and disruption of daily routines.

Both interventions comprise five two-hour sessions held weekly. While SH+ is offered in a group format, PM+ is conducted individually. The facilitators for both programs are trained, non-specialist individuals working under professional supervision. The target groups for both interventions include adults experiencing psychological distress.

Facilitators who delivered interventions were professional staff, including case managers, psychologists, and nurses, with basic mental health expertise. They received structured training and supervision from senior mental health professionals, certified PM+ and SH+ trainers, and supervisors to ensure fidelity to the WHO guidelines. Adherence to the WHO guidelines was monitored using several methods. These included supervision, tracking session attendance and completion rates, monitoring dropouts, and conducting fidelity checks. Reasons for non-attendance or discontinuation were documented. Supervisors observed a subset of sessions to ensure the intervention protocols were followed correctly.

***Eligibility criteria for the semi-structured interviews*** included such elements as: 1) participation in the OAT program; 2) presence of mental health issues (depression or anxiety symptoms) during the baseline screening; 3) confirmation of informed consent to participate in the interview; 4) participation in one of the piloted interventions: SH+ or PM+.

### Recruitment geography

Participants were recruited from three regions of Ukraine at designated healthcare institutions (OAT centers): Vinnytsia (Central Ukraine), Lviv (Western Ukraine), and Sumy (Eastern Ukraine). All monthly meeting notes with the staff were sampled for the study (7 notes).

### Ethical issues

The study protocol was developed in close collaboration with representatives from the OAT centers. Experts from the centers evaluated all research tools. The research team adhered to the Declaration of Helsinki and the Ethical Regulations of the National Psychological Association of Ukraine. The study protocol received approval from the Institutional Review Board of the State Institution “Public Health Centre of the Ministry of Health of Ukraine,” approval number 326. All participants signed the Informed Consent form before participating in the study interventions.

### Data collection methods and instruments

Monthly meetings were held with the OAT staff responsible for delivering the interventions to ensure the quality of all procedures and timely reactions to potential issues. Meeting notes were collected for the analysis from April to October 2023 (a total of 7 notes).

Semi-structured interviews were conducted with 12 clients in October 2023 (6 of whom participated in SH+ and 6 in PM+, approximately 3 months after the last sessions), based on the interview guide and consisting of such questions:

- *Could you please provide a brief description of your mental health condition?*
- *How would you describe your experience participating in the Problem Management Plus/ Self-Help Plus program?*
- *Have you noticed how this program has affected your condition? What sort of impact did it have exactly?*
- *What did you like about the program?*
- *What did it teach you?*
- *What disadvantages do you see in this program?*
- *Do you believe that such a program should be available in all OAT centers?*

All meetings with OAT staff and interviews were held online using Zoom, with audio recordings and further transcriptions for data analysis.

### Units of study

The study units included participants (staff and clients), documents (meeting notes and interview recordings), and events (staff meetings and interviews with clients).

#### Participants.

Clients of OAT Programs (12 clients participated in the study). They were undergoing opioid agonist treatment and had mental health symptoms (e.g., depression, anxiety). Six participated in SH+, a group-based intervention. Six participated in PM+, an individual intervention. Clients were recruited from three OAT centers in different regions of Ukraine: Vinnytsia (Central Ukraine), Lviv (Western Ukraine), Sumy (Eastern Ukraine). Clients attended five intervention sessions and were interviewed three months after completion. They shared their perspectives on the intervention experience, engagement, and perceived impact on their mental well-being.

#### Participants.

*OAT Staff.* The study involved an OAT center staff members (4–6 per center). Staff included psychologists, social workers, case managers, and nurses responsible for delivering SH+ and PM+. They received specialized training and supervision to implement WHO interventions. Staff facilitated intervention sessions and participated in monthly staff meetings (seven in total). Provided insights into intervention feasibility, recruitment challenges, and client engagement.

*Documents: Meeting Notes* (N = 7). Collected from April to October 2023 during monthly staff meetings. Contained staff observations on implementation challenges, client engagement strategies, and intervention outcomes.

*Documents: Interview Recordings* (N = 12). Semi-structured interviews were conducted with all 12 client participants in October 2023. Recorded via Zoom, transcribed.

*Events: Staff Meetings* (N = 7). Monthly coordination meetings with OAT staff were held to discuss intervention progress, barriers, and adjustments.

*Events: Interviews with Clients* (N = 12). Conducted three months after intervention completion to assess clients’ experiences, perceived benefits, and challenges.

### Data processing

Interviews were recorded via Zoom and transcribed manually. Meeting notes were taken during the meetings. The confidentiality and anonymity of participants were maintained.

### Data analysis

Thematic analysis was employed to analyze responses. Data were analyzed using thematic analysis following Braun and Clarke’s general principles [19]. After familiarization with the transcripts, we conducted inductive coding to capture participants’ experiences, complemented by deductive coding guided by the study focus on acceptability. Codes were clustered into themes through constant comparison, and themes were refined to ensure coherence and clarity. Both explicit (semantic) and underlying (latent) meanings were considered. To enhance rigor, two researchers reviewed coding decisions and theme development, resolving differences through discussion. Themes were selected based on their relevance to understanding how clients perceived and experienced the SH+ and PM+ interventions.

Staff data were derived from monthly coordination meeting notes that documented operational challenges, recruitment issues, scheduling adjustments, and facilitators’ observations during program delivery. These notes were designed as implementation monitoring tools rather than narrative qualitative data. For this reason, they were summarized descriptively and used to contextualize the implementation of SH+ and PM+, but they were not subjected to thematic analysis.

### Techniques to enhance trustworthiness

Detailed notes from meetings and recorded interviews were reviewed for consistency. The data were cross verified through staff feedback, participant interviews, and observational meeting notes. Researchers acknowledged their assumptions, biases, and power dynamics to minimize their influence on findings.

## Results

### Acceptability of the interventions by the clients of the OAT centers

A total of 12 clients participated in the interviews: 6 who completed SH+ and 6 who completed PM+. The sample included both men and women (8 men, 4 women), with ages ranging from 32 to 48 years. Participants were recruited from three OAT centers located in Vinnytsia, Lviv, and Sumy.

#### Acceptability of the SH+ intervention by the OAT clients.

Despite initial discomfort with the format, the SH+ intervention was well-accepted by OAT clients, primarily due to its supportive group environment and the practical self-help techniques it provided. The participants found value in the communication and emotional support within the group, as well as in the breathing and grounding exercises that they could apply beyond the sessions. This suggests that while adaptation to the intervention may take time, its long-term benefits in stress management and emotional regulation make it a valuable resource.

One key theme that emerges is the **initial discomfort with the SH+ format**, particularly due to the use of audio recordings and the group setting. Several participants described the experience as *“strange at first”* and mentioned feeling *“uncomfortable the first two meetings.”* However, this discomfort gradually diminished as they became accustomed to the format. The statement *“SH+ was strange at first, but as I walked, I realized it worked.”* highlights the process of adaptation and eventual acceptance.

Another important theme is the **value of communication and support within the group**. Many participants emphasized that one of the most beneficial aspects of SH+ was the opportunity to share their experiences, receive advice, and feel understood. Statements such as *“I liked communicating with people that someone is worried about us.”* and *“Most of all, I liked the atmosphere of understanding and communication.”* suggest that emotional support played a crucial role in making the intervention effective. The confidential nature of the group was also noted as a positive aspect, reinforcing trust and openness.

A third key theme is the **practical application of self-help techniques** learned during the intervention. Many participants reported using the breathing and grounding exercises in their daily lives. Statements such as *“I remember my breathing. I do it at home, and I can calm myself down.”* and *“Technique, if it gets bad, then find three objects around.”* indicate that these techniques were not only understood but also actively incorporated into coping strategies. The reference to *“writing problems on paper and throwing them away”* suggests that symbolic exercises were also impactful in helping participants manage stress.

Additionally, some participants highlighted the **overall usefulness of the intervention**, with several recommending it to others. Statements like *“I have recommended it to many; it helps.”* and *“It was useful; there were moments when I had to use it.”* suggest that the SH+ intervention provided tangible benefits in their daily lives.

#### Acceptability of the PM+ intervention by the OAT clients.

The PM+ intervention was well-accepted by OAT clients, largely due to its individual format, which fostered a sense of safety, confidentiality, and personal attention. The structured approach to skill-building, particularly in problem-solving, goal-setting, and emotional regulation, was highly valued. Participants not only learned practical coping techniques but also actively applied them in their lives, suggesting that the intervention had a lasting positive impact. The emphasis on privacy and trust in one-on-one sessions contributed to a more open and engaging experience, making PM+ a well-suited intervention for this population.

One key theme that emerges is **comfort with the individual format**. Unlike the initial discomfort some participants felt with the group-based SH+ intervention, PM+ was seen as familiar and reassuring. Statements such as *“I liked the individual work. Listened. Communication. The atmosphere is relaxed.”* suggest that participants appreciated the one-on-one format, which allowed for personal attention and open communication. The individual nature of the sessions provided a sense of control and structure, making it feel less like an obligation and more like a meaningful engagement.

A significant theme is **the importance of anonymity and confidentiality**. This aspect was mentioned more frequently than in SH+, indicating that participants placed a high value on privacy. The statement *“Most of all, I was supported by everything being anonymous. No one will know anything about me.”* underscores the reassurance that confidentiality provided. The ability to openly share without fear of judgment or disclosure appeared to be crucial for participants to fully engage in the intervention.

Another strong theme is **practical skill development**, particularly in problem-solving, goal-setting, and emotional regulation. Many participants highlighted the usefulness of techniques they learned, including creating schedules, setting realistic goals, and managing emotions. Statements like *“The most important thing was to restrain anger.”* and *“I liked the fact that I was able to write functions for the day.”* reflect an appreciation for structured planning and self-regulation. The ability to break tasks into achievable steps was particularly beneficial for those with physical or psychological challenges.

**Emotional self-regulation and coping techniques** were also widely valued. Many participants recalled learning breathing exercises, stress-relief methods, and strategies to manage depressive thoughts. Statements such as *“I remember the breathing technique to control myself in stressful situations.”* and *“Breathing, counting to 10, and trying to distract myself with something, imagining myself at sea.”* indicate that these techniques were not only taught but also actively used in daily life. The mention of *“showing calendars, how to relieve stress, how to drive away depressive thoughts”* suggests that participants appreciated structured guidance on managing their mental health.

#### Mental health prior to participation in interventions.

Answering the questions about their mental health state, clients noted that they had poor mental health, an unbalanced emotional state, a loss of meaning in life, deteriorated relationships with family, and a lack of control over their own behaviour and emotional sphere.

One dominant theme in these statements is **emotional suffering and despair**. The expressions of sadness and hopelessness, such as *“Everything was very sad”* and *“I did not see the point”*, suggest deep psychological distress. The statement *“There was a state of a broken person”* illustrates the sense of emotional defeat, indicating symptoms of depression and a loss of personal agency.

Another recurring theme is the **psychological impact of war and uncertainty**. The references to war, such as *“Especially when the war began, it was very difficult”* and *“The war began, panic periodically, emotional outbursts”*, reflect the distress and anxiety caused by external conflict. War creates a state of unpredictability and insecurity, which amplifies emotional struggles. The instability of the situation contributes to a constant sense of fear and panic, making it difficult for individuals to maintain emotional balance.

A significant theme that emerges is the **loss of motivation and purpose**. Several statements express an inability to engage in daily life activities, such as *“I did not know what would happen tomorrow, I do not want to work”,* and *“I cannot do anything at home”*. The phrase *“I lived like a zombie”* metaphorically represents a state of emotional numbness, where the individual feels detached from life.

**Heightened anxiety and nervousness** also appear as key emotional struggles. The statement *“You are nervous all the time, and you have many problems all the time”* indicates chronic stress, while *“No matter what word you say, you begin to break down”* highlights emotional fragility.

Another theme present in the statements is **physical and psychological fatigue**. The mention of fluctuating sleep patterns - *“Then I did not want to do anything, then insomnia, then I wanted to sleep”* - suggests emotional exhaustion.

Finally, **social disconnection and loneliness** play a crucial role in the emotional distress expressed. The statement *“My son grew up and lives separately; the question is, why live?”* indicates a deep sense of isolation and lack of social support. The loss of meaningful connections may intensify feelings of emptiness, contributing to the overall distress.

#### Mental health after the participation in interventions.

After participating in the pilot interventions, all clients, regardless of the type of intervention, noted a significant improvement in their condition, the establishment of the emotional sphere, and the harmonization of relationships with loved ones.

An important theme in these statements is **regaining emotional control and stability**. Several individuals mention improved emotional regulation, such as *“Now I am trying to control myself”*, *“I normalized my emotions. Control them.”*, and *“I have learned to restrain myself; that is the most important thing.”* These reflections highlight a conscious effort to manage emotions, suggesting personal growth in emotional resilience. Statements like *“I became calmer and more controlled”* and *“I have become calmer, a lot.”* reinforce this theme, indicating an increased ability to handle stress and emotional triggers.

Another significant theme is **improved mental and physical well-being**. Many statements reflect an overall improvement in health and quality of life. Expressions such as *“Now I feel good. I sleep great, have a good appetite, and I do not have panic attacks.”* and *“Sleep has improved. I want to live.”* illustrate positive changes in sleep patterns, appetite, and overall mental stability. The mention of *“Before PM+, many things were different. Work, daily routine, sleep, appetite, everything is fine now.”* suggests that structured support has played a key role in restoring balance in daily life.

A crucial aspect of recovery reflected in the statements is **the importance of social support and communication**. Statements like *“My relationship with my children and wife improved.”* and *“My relationship with my father changed.”* indicate the positive impact of emotional healing on family dynamics. Moreover, the benefits of social interactions are emphasized in *“While there were meetings, it was good; the fact that I had to come was already easier.”* and *“When communicating, it is much easier.”* These reflections suggest that structured programs, therapy, and social engagement contribute significantly to emotional recovery. However, challenges in communication persist for some individuals, as seen in *“At home, the situation is such that when someone calls an acquaintance, I do not pick up the phone to avoid communicating.”* This indicates that while progress has been made, social withdrawal remains a struggle for some.

A recurring theme is **renewed purpose and motivation for life**. Statements such as *“I began to live more fully. I feel the influence; I want to live.”* and *“I began to look at the world differently; I wanted to live.”* reveal a transformation from previous despair to a more hopeful outlook. The phrase *“Everything changed a little bit after class.”* suggests that structured interventions helped shift perspectives and encouraged individuals to re-engage with life. Additionally, statements like *“I started setting realistic goals, which are not something that I cannot do.”* indicate personal development and a focus on achievable progress.

Another important theme is **coping strategies and emotional resilience**. Statements such as *“You will explain to me such moments that I could not even think that you can calm and control yourself with ordinary breathing and activities.”* highlight the effectiveness of learned coping mechanisms. Similarly, *“I know how to behave and not panic in different situations.”* indicates that individuals have developed strategies to manage stress and anxiety more effectively. These insights suggest that individuals are not only recovering but also acquiring tools for long-term emotional well-being.

### Implementation context: Insights from OAT staff

The staff‑related material presented here reflects implementation insights derived from monthly coordination summaries rather than interview‑based qualitative data. These observations are included to provide contextual understanding of how SH+ and PM+ were delivered across sites; they should not be interpreted as thematic findings on acceptability.

#### Challenges and strategies in client recruitment and engagement.

Recruiting clients for the SH+ group intervention proved to be a significant challenge. Staff reported that forming and engaging groups required substantial effort, as clients were initially hesitant about group participation. In contrast, Problem Management Plus was easier to introduce on an individual basis. To overcome recruitment barriers, some sites initiated SH+ groups with just 2–3 participants. Over time, as information about the interventions spread among clients, internal demand increased, and clients began actively seeking participation.

#### Scheduling and attendance management for SH+.

Finding suitable schedules for SH+ sessions was another key challenge, with different sites adopting various approaches. Strategies included:

- Conducting smaller groups (2–3 participants) for increased flexibility.
- Scheduling sessions early in the morning (e.g., from 07:00) to coincide with clients’ arrival for medication.
- Maintaining a stable, fixed schedule with open participation to accommodate missed sessions.
- Offering sessions at varying times to ensure accessibility for working clients.

Unstable participation due to personal circumstances also posed a challenge. A practical solution was the introduction of multiple groups on a fixed schedule, allowing participants to attend alternative sessions if they missed their designated one.

#### Client adaptation and participation in SH+ and PM+.

Clients initially found the SH+ group format unfamiliar, often displaying confusion during the first session. This adaptation phase was shorter in the PM+ individual format due to the direct dialogue between clients and specialists. However, by the third SH+ session, engagement and motivation significantly improved.

During SH+ sessions, facilitators needed to adapt to clients’ learning paces. When information was delivered too quickly, it was necessary to slow down, pause audio materials, and provide additional clarifications. Despite an initial recommendation of 30 participants per group, staff reported that a group size of approximately six participants was optimal, as larger groups led to distractions and reduced engagement.

#### Factors influencing engagement and effectiveness.

Several factors were emphasized regarding the engagement and effectiveness of the interventions. First of all, the Social Dynamics. Groups where participants knew each other (e.g., couples, friends, or partners) were more stable and engaged compared to those where members were unfamiliar. Second, the Incentives. Providing minimal encouragement, such as coffee, tea, or snacks, had a noticeable positive impact on engagement and attendance. And third, the Perceived Passivity. Clients in SH+ groups appeared passive, often not actively participating in discussions. However, they later reported practicing techniques at home, indicating that passive participation did not necessarily equate to disengagement.

#### Client feedback and perceived impact.

Clients’ feedback and reactions to the interventions were often discussed during the meetings: clients consistently reported positive experiences with the interventions. Many described applying learned techniques in their daily lives, and experiencing improvements in their mental well-being and relationships. Additionally, participation in these interventions increased their awareness of other available services at OAT sites, such as medical consultations, social work support, and legal assistance.

*Summarizing*, both interventions were perceived by staff as having unique advantages, alongside with several implementation challenges. SH+ allowed for the simultaneous engagement of multiple clients, making it a scalable option for reaching larger populations. PM+ facilitated deeper individual engagement, allowing for personalised interactions, more flexible scheduling, and better identification of client needs, including referrals for social and legal support services. Psychologists and psychotherapists found PM+ easier to implement as it aligned more closely with traditional therapeutic approaches. Additionally, PM+ enabled a more thorough assessment of clients’ psychosocial needs, leading to referrals for additional services.

## Discussion

This study explored the acceptability of WHO’s scalable psychological interventions, SH+ and PM+, among clients and staff of opioid agonist treatment (OAT) programs in Ukraine. The findings provide important insights into the feasibility of implementing these interventions in a conflict-affected setting and highlight factors that influence client engagement and program effectiveness.

The results indicate that participants generally received both interventions well, though they presented distinct implementation challenges and benefits. SH+ fostered a sense of community and peer support, which participants found valuable. However, initial engagement was difficult due to unfamiliarity with the group format and audio-guided sessions. In contrast, PM+ was perceived as more accessible and flexible, particularly for clients with chaotic life styles. The individual format of PM+ facilitated stronger rapport-building, allowing for a personalized approach that resonated with participants.

Nonetheless, as SH+ sessions progressed, participants reported significant benefits from the group-based approach, suggesting that initial reservations may be mitigated through continued participation and familiarity with the intervention structure. One of the key strengths of SH+ was its ability to create a structured and safe environment where clients could share their experiences and develop coping mechanisms collectively. The group format facilitated the normalization of distress and fostered social connectedness, a crucial aspect for individuals experiencing isolation due to opioid use disorder (OUD). Despite initial hesitations, participants who attended multiple sessions expressed increased motivation and appreciation for the intervention’s structured nature.

Conversely, PM+ allowed for a more tailored approach to addressing individual psychological distress. Clients reported that the flexibility of scheduling PM+ sessions was particularly advantageous, given their varying daily commitments and unpredictable life circumstances. The intervention’s focus on problem-solving strategies and emotional regulation was seen as practical and immediately applicable, reinforcing previous findings on the efficacy of PM+ in conflict-affected populations [20].

The study’s results on PM+ align with similar studies conducted in other countries (Kenya, Ethiopia, Syria, and Honduras), revealing the overall acceptability of the PM+, perceiving its strategies as meaningful [21], relevant, appropriate [22], and acceptable [23].

Despite the positive reception, several barriers to implementation were identified. For SH+, the challenge of initial client engagement highlights the need for enhanced recruitment strategies. Many participants were unfamiliar with the concept of structured psychological interventions and required additional motivation to attend sessions. Strategies such as client education, peer endorsements, and tangible incentives (e.g., refreshments during sessions) proved useful in improving attendance and engagement.

While the individualized format was well received for PM+, logistical constraints posed challenges, such as the limited availability of trained facilitators and scheduling difficulties. Some participants reported difficulty maintaining consistent attendance due to external stressors such as displacement, financial instability, and the ongoing war. These barriers underscore the need for additional resource allocation, including increased facilitator training and the integration of remote or digital adaptations where feasible. Again, such results correlate with similar findings from other countries, highlighting challenges with setting up the session and poor commitment of people living with HIV [21], or non-sustainability of the non-specialist health workforce in rural areas of Kenya [23]. However, participants with OUD were not part of these studies on PM+ and SH+, and our findings suggest that scalable psychological interventions have the potential to enhance mental health support in OAT programs (particularly in Ukraine).

Participants also described ways in which the interventions complemented the existing services offered at OAT centers. Several clients noted that attending SH+ or PM+ sessions increased their engagement with the OAT site itself and made them more aware of available support services, including medical follow‑up, counseling, and social or legal assistance. For some, the interventions acted as an additional point of contact with supportive staff, strengthening trust and continuity of care. Staff also observed that SH+ and PM+ fit naturally into the routine structure of OAT services, as most clients already visited the centers regularly for medication and case‑management activities. This made scheduling feasible and allowed facilitators to identify and support clients experiencing acute distress.

Findings indicate that SH+ and PM+ complement OAT services by addressing psychological distress that often interferes with treatment stability and recovery. Because OAT centers function as regular points of contact for clients, they provide an appropriate and accessible setting for delivering brief psychosocial interventions. The familiarity of the environment, combined with established rapport with staff, facilitated trust and encouraged participation. Moreover, clients reported that involvement in the interventions increased their awareness of (and willingness to seek) additional services available at the centers. These observations suggest that integrating scalable psychological interventions into OAT programs may strengthen holistic care and improve clients’ overall functioning and well‑being.

The Ukrainian wartime context further underscores the relevance of SH+ and PM+ for OAT clients. The ongoing war has increased exposure to traumatic events, disrupted social networks, and created continuous uncertainty, all of which contribute to heightened psychological distress. SH+ and PM+ were specifically designed by WHO to support populations facing adversity, limited access to specialist care, and chronic stress, making them highly suitable for delivery in conflict‑affected settings. The interventions’ focus on emotional regulation, problem‑solving, and stress‑management provided participants with practical skills to cope with war‑related stressors and maintain stability in daily functioning. Their brief format and reliance on trained non‑specialists also make them feasible within a health system strained by displacement, staff shortages, and fluctuating resources.

## Limitations of the study

This study has several limitations. Recruitment of interview participants proved challenging, as some clients were reluctant to engage in in‑depth discussions about their mental health due to stigma, limited trust, and the ongoing war‑related stressors. Security concerns, displacement, and unstable living conditions further constrained participation and contributed to the relatively small sample size.

Additionally, interviews were conducted several months after completion of the interventions, which may have influenced participants’ recall of their experiences. While the qualitative design allowed for in‑depth exploration of perceptions and experiences, the findings should be interpreted as context‑specific and illustrative rather than representative. Future research could build on these findings by including additional sites, or complementary quantitative measures.

## Conclusions

The implementation of SH+ and PM+ within OAT programs in Ukraine demonstrates promising potential for addressing the mental health needs of individuals with OUD. While both interventions were acceptable to participants, their effectiveness was influenced by factors such as familiarity with the intervention format, logistical challenges, and individual preferences. To maximize impact and increase sustainability, further training of OAT staff alongside continued professional supervision should be considered. Future studies should focus on optimizing intervention delivery, assessing long-term effectiveness, and exploring digital adaptations to improve accessibility in conflict-affected settings.

In the context of the ongoing war, these WHO scalable interventions offer an especially suitable approach for addressing heightened psychological distress, supporting resilience, and expanding mental health support within overstretched OAT services.

By addressing these challenges and leveraging the strengths of both interventions, OAT programs in Ukraine can play a crucial role in bridging the mental health treatment gap, ultimately improving outcomes for individuals struggling with opioid use disorder and psychological distress.

## Supporting information

S1 Checklist The SRQR reporting checklist. (DOCX)
