# Supplementary material for: Acceptability of Self-Help Plus and Problem Management Plus interventions among clients and implementation insights from staff of Opioid Agonist Treatment programs in Ukraine
Source: PLOS Ment Health. 2026 Jun 22;3(6):e0000470. doi: 10.1371/journal.pmen.0000470 (PMC13286228; doi:10.1371/journal.pmen.0000470)
Supplement: Attachment — Submitted filename: Responce to Reviewers 2.docx [file pmen.0000470.s004.docx]

Dear Reviewers,

Thank you very much for the feedback on our manuscript and for the further comments you provided. We have addressed all comments point‑by‑point and revised the manuscript accordingly. We hope that the revised version adequately reflects your suggestions and that the manuscript is ready for production.

| **Reviewers` Comments** | **Authors` Reponses** |
| --- | --- |
| Reviewer #2 |  |
| • One of the instances of ‘the’ in the title should be removed, i.e., Acceptability of Self-Help Plus and Problem Management Plus interventions among staff and by clients and implementation insights of from the staff of the Opioid Agonist Treatment programs in Ukraine | “the” are corrected:  Acceptability of Self-Help Plus and Problem Management Plus Interventions among Clients and Implementation Insights from Staff of Opioid Agonist Treatment Programs in Ukraine |
| • Under Materials and Methods, the paragraph starting ‘Phenomenology’ needs one or more references to validate/support the claims being made here. | References now added |
| • The ‘Limitations’ section needs to be before the ‘Conclusions’ | Limitation now moved before the Conclusion |
| • The ‘Sampling strategy’ header should be removed as it is not relevant here. | Removed |
| • Consider presenting the eligibility criteria in sentence rather than dot point format. | Now presented in a sentence |
| • In the discussion, the sentences starting “Again, such results…” and “However, participants…” should be linked together (i.e., as one paragraph) and to the paragraph that precedes it which starts “While the individualized format…” | All elements are linked now at one paragraph |

Authors
