# Supplementary material for: Acceptability of Self-Help Plus and Problem Management Plus interventions among clients and implementation insights from staff of Opioid Agonist Treatment programs in Ukraine
Source: PLOS Ment Health. 2026 Jun 22;3(6):e0000470. doi: 10.1371/journal.pmen.0000470 (PMC13286228; doi:10.1371/journal.pmen.0000470)
Supplement: Attachment — Submitted filename: Responce to Reviewers.docx [file pmen.0000470.s003.docx]

Dear Reviewers,

Thank you very much for the careful reading of our manuscript and for the constructive comments you provided. We appreciate the time and expertise that went into this review process. Your feedback helped us identify several areas where the manuscript could be strengthened and clarified.

We have addressed all comments point‑by‑point and revised the manuscript accordingly. In many sections, we expanded the background and methodological explanations, clarified the study objectives, added missing details, and restructured parts of the Results and Discussion to improve the overall narrative. Below, we outline the changes made in response to each comment and indicate where these revisions can be found in the updated manuscript.

We hope that the revised version adequately reflects your suggestions and that the manuscript is now clearer, more complete, and more informative.

| **Reviewers` Comments** | **Authors` Reponses** |
| --- | --- |
| Reviewer #1 |  |
| INTRODUCTION  The authors explain that their study aimed to explore the acceptability of psychological interventions in participants of OAT programs in Ukraine, but they do not really explain what these psychological consist of, and what are their objectives. | We agree, we have revised the Introduction. Specifically, we added a description of SH+ and PM+ |
| The last sentence citing the SRQR recommendations should be placed at the beginning of the Materials & Methods section. An item checklist should be added into the supplemental materials | Sreference to SRQR now placed at the beginning of the Materials & Methods section.  SRQR checklist is added as supplemental materials |
| MATERIALS & METHODS  - Understanding the exact objective of the survey is a bit difficult. The authors explain first that it is to “study the acceptability” of WHO psychological interventions. The next sentence, it is stated that the authors wished “to understand how experiences and contextual factors influence the perceptions of OTP centers’ staff and clients of the interventions”. To me, both objectives are different. Can the authors be clearer on the survey objectives? | Thank you for this observation. In response, we have clarified and streamlined the description of the study aims in both the Introduction and Methods sections |
| - The authors could use the PICO framework to better explain their research approach. https://www.library.sydney.edu.au/support/searching/pico | Using the PICO framework across the SRQR frame could create a several repetitions in the text, so we decided to stick to the SRQR checklist |
| - The acronyms SH+ and PM+, apparently related to WHO interventions, are introduced in the M&M section but, unless I am mistaken, they are not detailed at all throughout the manuscript, which is a bit confusing for the reader. I think that the Introduction section should explain what these interventions are, and what they aim at. At this stage, it is a major issue that these programs be not explained. | We added a general description of SH+ and PM+ to the Introduction section, and left detailed description in the M&M section. |
| - Several important contextual pieces of information pop up suddenly in the manuscript whereas they should have been explained much earlier. For example, page 6, amid the methodological aspects of the survey, the authors report that their qualitative survey is “a part of a pragmatic trial assessing the effectiveness of the SH+ and PM+ interventions for individuals with OUD…”. At this point, the reader understands that there is a quantitative study associated with their qualitative survey. This element should have been explained much earlier, for example in the last part of the Introduction section, or in the beginning of the M&M section. | Thank you for noticing, now as trial data are published, we added relevant information to the Introduction section. |
| - The WHO interventions (SH+ and PM+) are finally explained page 6 in the M&M section. This is very unusual. Again, I recommend to explain the context IN THE INTRODUCTION SECTION OF THE MANUSCRIPT. Readers would appreciate external references, for example, to know if these WHO interventions have already been implemented and evaluated elsewhere. | We added a general description of SH+ and PM+ to the Introduction section, and left detailed more contextual description in the M&M section. |
| RESULTS  - page 10: “Recruiting clients for the Self-Help Plus group”. The SH+ abbreviation has been used throughout the previous text (and never defined) and then suddenly the full name of the intervention is used. Please fulfill basic rules of the scientific writing, i.e., defining acronyms when use first, and then only use it throughout the rest of the text. | Throughout the text we made the corrections |
| - In the “Acceptability of the interventions by the OAT centers` staff” subsection of the Results, I am surprised to find no verbatim at all. Why? | Thank you for this observation. We agree that the absence of verbatim quotations in this subsection may appear unusual. The reason is methodological:   - The staff‑related findings were derived not from individual qualitative interviews, but from monthly coordination meeting notes, which served primarily as implementation monitoring documents rather than narrative datasets. - These notes contained summarized accounts of staff discussions rather than verbatim transcripts of their speech. Because facilitators did not record full conversational exchanges, but instead produced concise summaries of challenges, decisions, and observations, there were no original verbatim quotations available that would meet qualitative reporting standards. - For this reason, the staff findings were presented as thematic summaries instead of quote‑supported themes. |
| - In the “Acceptability of the interventions by the clients of the OAT centers” subsection, the authors report pre-post intervention verbatims of patients expressing their psychological improvement. I am confused, as the authors explained in the M&M section that they aimed to explore the “acceptability” of the interventions, whereas they seemed to explore the effectiveness of the interventions. | Thank you for this important observation. We agree that some of the participants’ statements describing perceived psychological improvements may appear similar to effectiveness outcomes. However, we would like to clarify how these statements relate specifically to acceptability rather than to the effectiveness of the interventions.  The purpose of the interviews was not to evaluate clinical effectiveness, but rather to understand how clients experienced the interventions and what aspects contributed to or hindered their acceptance. In qualitative research, expressions of perceived improvement often emerge naturally when participants discuss why they found an intervention helpful, meaningful, or worth recommending.  In this context:  Participants’ descriptions of improved mood, sleep, emotional regulation, or functioning were interpreted as indicators of perceived usefulness, which is a core component of acceptability.  These verbatim statements illustrate why clients valued the interventions, why they were willing to participate, and why they would recommend them to others.  We do not claim or measure clinical effectiveness, and we acknowledge that these perceptions do not constitute outcome evidence |
| DISCUSSION  - In practice, the authors report qualitative data suggesting that WHO psychological interventions are correctly accepted and apparently effective (based on qualitative data) to improve the psychological status and global functioning of participants, but when reading the discussion, I am wondering how these interventions are related to OAT programs. They could have been proposed to any type of population, and the impact of the interventions on the OAT programs was not explored, was it? | Thank you for the feedback, we added a notion that these other studies not were conducted with participants having OUD. |
| ABSTRACT  - Even unstructured, the abstract should follow an IMRAD structure (unless the journal expressly recommends otherwise). Therefore, an introductory couple of sentences explaining what the interventions entail would be welcome. | Thank you for this helpful suggestion. We fully agree that the abstract lacked introductory context and did not clearly follow an IMRAD‑like structure. To address this, we have revised the beginning of the abstract to briefly describe the nature and purpose of the WHO interventions (SH+ and PM+) |
| - Similarly, there is no conclusion in the abstract, which ends abruptly with a result sentence. This is very unusual. | Thank you for pointing this out. We agree that the abstract ended too abruptly and did not include a concluding statement. To address this, we have added a brief conclusion summarizing the overall implications of the findings |
| - Please do not use “paper” but “survey” | We changed to “study” as surveys were not used in this study design |
| Reviewer #2: |  |
| More information about the programs and their intended use is needed, i.e., were they intended to help support people’s mental health generally, or specifically in relation to their drug use or treatment experiences, and/or the impacts of living during a time of war (or both)? Greater clarity would assist in understanding the results reported. | Thank you for this important comment. We agree that the initial manuscript did not fully clarify the intended purpose and scope of the SH+ and PM+ interventions in the context of this study. To address this, we have added a detailed explanation in the “Methods – Interventions subsection.” |
| Also, the staff data is not really examined or reported using thematic analysis techniques, and its is focused on implementation rather than acceptability per se, so it could be reported as supplemental rather than primary findings. | Thank you for this insightful and constructive comment. We agree that the staff‑related material differs methodologically from the client interview data. The staff data came from monthly coordination meeting notes, which summarize implementation processes, operational challenges, and practical observations rather than capturing verbatim, narrative, or phenomenological accounts.  Because these notes were created for program monitoring rather than for research purposes, they were not suitable for full thematic analysis and therefore should not be presented as equivalent to the interview‑based findings.  We have reframed the staff data as implementation‑focused contextual information, not as primary qualitative findings.  We have revised the Methods section to clarify that staff meeting notes were reviewed descriptively and used to contextualize the delivery of SH+ and PM+, not to analyze acceptability.  We have removed language implying that these data were thematically analyzed, and we explicitly state that the thematic analysis was applied only to client interviews.  In the Results, we now introduce the staff material with a clear explanation of its purpose (implementation context) to avoid confusion with acceptability‑related findings. |
| • More context is needed to justify why psychological support interventions are necessary for this consumer/population group, and why SH+ and PM+ were selected as being suitable to use/test in these settings. The goals in applying these interventions, and how they work in practice, also needs some description. Please add a little more background on these interventions (and perhaps the reason for applying them to this client group) in your introduction. | Thank you for this valuable comment. We agree that the Introduction needed clearer justification for why psychological support interventions are important for individuals receiving OAT in Ukraine, and why SH+ and PM+ are particularly suitable.  In response, we have expanded the Introduction. |
| • Methods: Consider reordering so that you start with the study design (i.e., the paragraph you have titled, ‘Context’ along with ‘Qualitative approach and research paradigm’), then continue with interventions, sampling, etc., with ‘Researcher’s characteristics and reflexivity’ at the end of this section (as it references considerations that need an understanding of the methods). | In this case we followed the SRQR reporting guideline, proposing the order of the elements as presented in the article. |
| • Under Methods – Qualitative approach and research paradigm, more detail is needed about the selected phenomenological approach and interpretive paradigm, including a few sentences that describe what these involve and how they were applied in this study. | Thank you for this insightful comment. We agree that the description of the phenomenological approach and interpretive paradigm was too brief. To address this, we have expanded this subsection. |
| • Under methods – Sampling strategy: In this section describe the sampling strategy for interview participants, moving any discussion of the problems in this to the ‘Limitations’ section (that is, after/ at the end of the discussion section) and identifying the number sampled under ‘Participants’ or at the start of the results section. | The problem discussion now moved to the Limitation section. |
| • Results: It is not clear which of the findings are themes. Perhaps include a figure or table that provides an overview of the thematic structure by participant group, theme and subthemes, etc.  • Acceptability is given as an overarching theme, but the subthemes do not necessarily relate to intervention acceptability (i.e., identifying problems in client recruitment, scheduling, and participation are more about implementation and aren’t clearly linked to intervention acceptability). Consider restructuring or removing the broader theme of acceptability in the results, using it instead as a frame of reference for the discussion only. | Thank you, we restructured the Results section, to focus on the aceptability themes and to make them more salient. |
| • The staff data is a summary of meeting discussions and does not really comply with a qualitative thematic analysis method. My suggestion would be to present it instead as a table (with a paragraph or two summarising the main points made by staff) and refer to this information as providing a contextual summary of program implementation experiences and advice, to situate the research findings generated from client interviews. I would then remove reference to ‘staff’ in the title and rewrite the methods to include the review of staff meeting recordings as implementation findings (i.e., not as part of the thematic analysis). | The part with the staff data now presented at the end of the Reulst section, framed as “Implementation Context: Insights From OAT Staff”  We also renamed an article: “Acceptability of Self-Help Plus and Problem Management Plus interventions by clients and implementation insights from the staff of the Opioid Agonist Treatment programs in Ukraine” |
| • The terminology of OAT as a ‘substitute’ medication is now dated, as it contributes to stigmatising notions of prescribed OAT as not being any different to heroin/illicit opioid consumption. (see this article: https://www.thelancet.com/journals/lancet/article/PIIS0140-6736(15)60750-4/fulltext ). Consider replacing references to ‘substitution’ with ‘treatment’. | Yes, great observation, we changed the wording |
| • In the second paragraph of the introduction, add a sentence or two that justifies the importance of providing OAT services (i.e., reduces overdose death, improves overall health and wellbeing, etc.) with reference to supporting literature. | Thank you, however, we do not want to overburden the text with the information, that is alerady well established and widely accepted. |
| • Under Methods – Interventions, provide the references for each of the interventions (i.e., 11 and 12 in your reference list) | Thank you, corrected |
| • Under methods – Data analysis: More information is needed here about the specific techniques used, possibly including references and noting what goals or principles guided theme selections and development. | Thank you for this helpful comment. We agree that the description of our data analysis process required additional detail. In response, we have expanded the Data Analysis subsection |
| • In the results, indicate how many interviewees participated in each program, and give an overview of interviewee demographic information (gender, age, location, etc). | Relevant information is added. |
| Also, if possible, aim to provide some findings that discuss thowthese programs complement OAT services, the suitability of these services as a program setting, etc., as these details may be important to readers. | Thank you for this valuable comment. We agree that highlighting how SH+ and PM+ complement OAT services would strengthen the manuscript. In response, we have expanded the Discussion section. |
| Discussion contains a misspelled word (resustyles) | Corrected |
| • Discussion and Conclusion should also refer to the appropriateness of these programs in responding to impacts from an ongoing war (not just in reference to implementation, as this function is reported in the results). | Thank you for this important comment. We agree that although the Results section highlights war‑related contextual factors influencing implementation, the Discussion and Conclusion did not sufficiently articulate why SH+ and PM+ are particularly appropriate interventions during wartime. In response, we have added new sentences to both the Discussion and Conclusion. |
| In the limitations, generalisability isn’t really appropriate to report on (as qualitative data is by its nature generally not intended to be generalisable), so remove reference to this. The problems with recruitment should be reported in this section. | Thank you for this important clarification. We agree that referring to generalisability was not appropriate for a qualitative study and appreciate the suggestion. In response, we have removed references to generalisability from the Limitations section.  We have also expanded the Limitations section to explicitly describe recruitment challenges. |
| In the limitations, generalisability isn’t really appropriate to report on (as qualitative data is by its nature generally not intended to be generalisable), so remove reference to this. The problems with recruitment should be reported in this section. | Thank you, the Limitation section was rewritten. |
| Reference 12 is missing ‘Problem Management Plus (PM+)’ from the start of the title. | In the WHO suggested citation the same issue, therefore we decided to follow the exact citation siggested by WHO. |
