# Supplementary material for: Acceptability of Self-Help Plus and Problem Management Plus interventions among clients and implementation insights from staff of Opioid Agonist Treatment programs in Ukraine
Source: PLOS Ment Health. 2026 Jun 22;3(6):e0000470. doi: 10.1371/journal.pmen.0000470 (PMC13286228; doi:10.1371/journal.pmen.0000470)
Supplement: Peer review history

# Peer review: Decision Letter 0

**DOI:** 10.1371/journal.pmen.0000470.r001

**Authors:** Karli Montague-Cardoso

5 Mar 2026

PMEN-D-25-00452

Acceptability of Self-Help Plus and Problem Management Plus interventions among staff and clients of the Opioid Agonist Treatment programs in Ukraine

PLOS Mental Health

Dear Dr. Klymchuk,

Thank you for submitting your manuscript to PLOS Mental Health and I am very sorry for the severe delay in reaching a decision. This was due to difficulties securing reviewers. Thank you for your patience. After careful consideration of the reviewer reports, we feel that your paper has merit but does not fully meet PLOS Mental Health’s publication criteria as it currently stands. Therefore, we invite you to submit a revised version of the manuscript that addresses the points raised during the review process.

Please address all comments, which you can find at the end of this email.

Please submit your revised manuscript by Apr 18 2026 11:59PM. If you will need more time than this to complete your revisions, please reply to this message or contact the journal office at mentalhealth@plos.org. When you're ready to submit your revision, log on to https://www.editorialmanager.com/pmen/ and select the 'Submissions Needing Revision' folder to locate your manuscript file.

Please include the following items when submitting your revised manuscript:

- A letter that responds to each point raised by the editor and reviewer(s). You should upload this letter as a separate file labeled 'Response to Reviewers'.
- A marked-up copy of your manuscript that highlights changes made to the original version. You should upload this as a separate file labeled 'Revised Manuscript with Track Changes'.
- An unmarked version of your revised paper without tracked changes. You should upload this as a separate file labeled 'Manuscript'.

Guidelines for resubmitting your figure files are available below the reviewer comments at the end of this letter.

We look forward to receiving your revised manuscript.

Kind regards,

Karli Montague-Cardoso

Staff Editor

PLOS Mental Health

Journal Requirements:

1. Please ensure that your Ethics Statement is available in its entirety at the beginning of your Methods section, under a subheading 'Ethics Statement'.

2. For studies involving third-party data, we encourage authors to share any data specific to their analyses that they can legally distribute. PLOS recognizes, however, that authors may be using third-party data they do not have the rights to share. When third-party data cannot be publicly shared, authors must provide all information necessary for interested researchers to apply to gain access to the data. (https://journals.plos.org/plosone/s/data-availability#loc-acceptable-data-access-restrictions)

For any third-party data that the authors cannot legally distribute, they should include the following information in their Data Availability Statement upon submission:

1) A description of the data set and the third-party source

2) If applicable, verification of permission to use the data set

3) Confirmation of whether the authors received any special privileges in accessing the data that other researchers would not have

4) All necessary contact information others would need to apply to gain access to the data

If the reviewer comments include a recommendation to cite specific previously published works, please review and evaluate these publications to determine whether they are relevant and should be cited. There is no requirement to cite these works unless the editor has indicated otherwise.

Additional Editor Comments (if provided):

[Note: HTML markup is below. Please do not edit.]

Reviewers' comments:

Reviewer's Responses to Questions

**Comments to the Author**

1. Does this manuscript meet PLOS Mental Health’s publication criteria? Is the manuscript technically sound, and do the data support the conclusions? The manuscript must describe methodologically and ethically rigorous research with conclusions that are appropriately drawn based on the data presented.

Reviewer #1: Partly

Reviewer #2: Partly

**********

2. Has the statistical analysis been performed appropriately and rigorously?

Reviewer #1: N/A

Reviewer #2: N/A

**********

3. Have the authors made all data underlying the findings in their manuscript fully available (please refer to the Data Availability Statement at the start of the manuscript PDF file)?

The PLOS Data policy requires authors to make all data underlying the findings described in their manuscript fully available without restriction, with rare exception. The data should be provided as part of the manuscript or its supporting information, or deposited to a public repository. For example, in addition to summary statistics, the data points behind means, medians and variance measures should be available. If there are restrictions on publicly sharing data—e.g. participant privacy or use of data from a third party—those must be specified.

Reviewer #1: Yes

Reviewer #2: No

**********

4. Is the manuscript presented in an intelligible fashion and written in standard English?

PLOS Mental Health does not copyedit accepted manuscripts, so the language in submitted articles must be clear, correct, and unambiguous. Any typographical or grammatical errors should be corrected at revision, so please note any specific errors here.

Reviewer #1: Yes

Reviewer #2: Yes

**********

5. Review Comments to the Author

Please use the space provided to explain your answers to the questions above. You may also include additional comments for the author, including concerns about dual publication, research ethics, or publication ethics. (Please upload your review as an attachment if it exceeds 20,000 characters)

Reviewer #1: INTRODUCTION

The authors explain that their study aimed to explore the acceptability of psychological interventions in participants of OAT programs in Ukraine, but they do not really explain what these psychological consist of, and what are their objectives.

The last sentence citing the SRQR recommendations should be placed at the beginning of the Materials & Methods section. An item checklist should be added into the supplemental materials

MATERIALS & METHODS

- Understanding the exact objective of the survey is a bit difficult. The authors explain first that it is to “study the acceptability” of WHO psychological interventions. The next sentence, it is stated that the authors wished “to understand how experiences and contextual factors influence the perceptions of OTP centers’ staff and clients of the interventions”. To me, both objectives are different. Can the authors be clearer on the survey objectives?

- The authors could use the PICO framework to better explain their research approach. https://www.library.sydney.edu.au/support/searching/pico

- The acronyms SH+ and PM+, apparently related to WHO interventions, are introduced in the M&M section but, unless I am mistaken, they are not detailed at all throughout the manuscript, which is a bit confusing for the reader. I think that the Introduction section should explain what these interventions are, and what they aim at. At this stage, it is a major issue that these programs be not explained.

- Several important contextual pieces of information pop up suddenly in the manuscript whereas they should have been explained much earlier. For example, page 6, amid the methodological aspects of the survey, the authors report that their qualitative survey is “a part of a pragmatic trial assessing the effectiveness of the SH+ and PM+ interventions for individuals with OUD…”. At this point, the reader understands that there is a quantitative study associated with their qualitative survey. This element should have been explained much earlier, for example in the last part of the Introduction section, or in the beginning of the M&M section.

- The WHO interventions (SH+ and PM+) are finally explained page 6 in the M&M section. This is very unusual. Again, I recommend to explain the context IN THE INTRODUCTION SECTION OF THE MANUSCRIPT. Readers would appreciate external references, for example to know if these WHO interventions have already been implemented and evaluated elsewhere.

RESULTS

- page 10: “Recruiting clients for the Self-Help Plus group”. The SH+ abbreviation has been used throughout the previous text (and never defined) and then suddenly the full name of the intervention is used. Please fulfill basic rules of the scientific writing, i.e., defining acronyms when use first, and then only use it throughout the rest of the text.

- In the “Acceptability of the interventions by the OAT centers` staff” subsection of the Results, I am surprised to find no verbatim at all. Why?

- In the “Acceptability of the interventions by the clients of the OAT centers” subsection, the authors report pre-post intervention verbatims of patients expressing their psychological improvement. I am confused, as the authors explained in the M&M section that they aimed to explore the “acceptability” of the interventions, whereas they seemed to explore the effectiveness of the interventions.

DISCUSSION

- In practice, the authors report qualitative data suggesting that WHO psychological interventions are correctly accepted and apparently effective (based on qualitative data) to improve the psychological status and global functioning of participants, but when reading the discussion, I am wondering how these interventions are related to OAT programs. They could have been proposed to any type of population, and the impact of the interventions on the OAT programs was not explored, was it?

ABSTRACT

- Even unstructured, the abstract should follow an IMRAD structure (unless the journal expressly recommends otherwise). Therefore, an introductory couple of sentences explaining what the interventions consist of would be welcome.

- Similarly, there is no conclusion in the abstract, which ends abruptly with a result sentence. This is very unusual.

- Please do not use “paper” but “survey”

Reviewer #2: This paper presents data summarising the acceptability of two interventions for supporting Ukrainian consumers of OAT programs during wartime, using data from consumers and program staff. The authors are to be commended for their efforts in conducting research during a time of conflict, as the data generated is novel and provides important information that can guide future implementation of psychological interventions in a wartime context. More information about the programs and their intended use is needed, i.e., were they intended to help support people’s mental health generally, or specifically in relation to their drug use or treatment experiences, and/or the impacts of living during a time of war (or both)? Greater clarity would assist in understanding the results reported. Also, the staff data is not really examined or reported using thematic analysis techniques, and its is focused on implementation rather than acceptability per se, so it could be reported as supplemental rather than primary findings. Overall, the study provides useful findings to support the further implementation of psychological support interventions in OAT service settings.

Major comments

• More context is needed to justify why psychological support interventions are necessary for this consumer/population group, and why SH+ and PM+ were selected as being suitable to use/test in these settings. The goals in applying these interventions, and how they work in practice, also needs some description. Please add a little more background on these interventions (and perhaps the reason for applying them to this client group) in your introduction.

• Methods: Consider reordering so that you start with the study design (i.e., the paragraph you have titled, ‘Context’ along with ‘Qualitative approach and research paradigm’), then continue with interventions, sampling, etc., with ‘Researcher’s characteristics and reflexivity’ at the end of this section (as it references considerations that need an understanding of the methods).

• Under Methods – Qualitative approach and research paradigm, more detail is needed about the selected phenomenological approach and interpretive paradigm, including a few sentences that describe what these involve and how they were applied in this study.

• Under methods – Sampling strategy: In this section describe the sampling strategy for interview participants, moving any discussion of the problems in this to the ‘Limitations’ section (that is, after/ at the end of the discussion section) and identifying the number sampled under ‘Participants’ or at the start of the results section.

• Results: It is not clear which of the findings are themes. Perhaps include a figure or table that provides an overview of the thematic structure by participant group, theme and subthemes, etc.

• Acceptability is given as an overarching theme, but the subthemes do not necessarily relate to intervention acceptability (i.e., identifying problems in client recruitment, scheduling, and participation are more about implementation and aren’t clearly linked to intervention acceptability). Consider restructuring or removing the broader theme of acceptability in the results, using it instead as a frame of reference for the discussion only.

• The staff data is a summary of meeting discussions and does not really comply with a qualitative thematic analysis method. My suggestion would be to present it instead as a table (with a paragraph or two summarising the main points made by staff) and refer to this information as providing a contextual summary of program implementation experiences and advice, to situate the research findings generated from client interviews. I would then remove reference to ‘staff’ in the title and rewrite the methods to include the review of staff meeting recordings as implementation findings (i.e., not as part of the thematic analysis).

Minor comments

• The terminology of OAT as a ‘substitute’ medication is now dated, as it contributes to stigmatising notions of prescribed OAT as not being any different to heroin/illicit opioid consumption. (see this article: https://www.thelancet.com/journals/lancet/article/PIIS0140-6736(15)60750-4/fulltext ). Consider replacing references to ‘substitution’ with ‘treatment’.

• In the second paragraph of the introduction, add a sentence or two that justifies the importance of providing OAT services (i.e., reduces overdose death, improves overall health and wellbeing, etc.) with reference to supporting literature.

• Under Methods – Interventions, provide the references for each of the interventions (i.e., 11 and 12 in your reference list)

• Under Methods – Participants: If possible, give a total number of staff participants and how many participated per meeting, as well as the dates and locations of meetings (possibly reporting this at the start of the results section instead).

• Under methods – Data analysis: More information is needed here about the specific techniques used, possibly including references and noting what goals or principles guided theme selections and development.

• In the results, indicate how many interviewees participated in each program, and give an overview of interviewee demographic information (gender, age, location, etc). Also, if possible, aim to provide some findings that discuss the ways that these programs complement OAT services, the suitability of these services as a program setting, etc., as these details may be important to readers.

• Discussion contains a misspelled word (resustyles)

• Discussion and Conclusion should also refer to the appropriateness of these programs in responding to impacts from an ongoing war (not just in reference to implementation, as this function is reported in the results).

• In the limitations, generalisability isn’t really appropriate to report on (as qualitative data is by its nature generally not intended to be generalisable), so remove reference to this. The problems with recruitment should be reported in this section.

• Reference 12 is missing ‘Problem Management Plus (PM+)’ from the start of the title.

**********

6. PLOS authors have the option to publish the peer review history of their article (what does this mean?). If published, this will include your full peer review and any attached files.

**Do you want your identity to be public for this peer review?**  If you choose “no”, your identity will remain anonymous but your review may still be made public.

For information about this choice, including consent withdrawal, please see our Privacy Policy.

Reviewer #1: **Yes:** Benjamin ROLLAND

Reviewer #2: No

**********

[NOTE: If reviewer comments were submitted as an attachment file, they will be attached to this email and accessible via the submission site. Please log into your account, locate the manuscript record, and check for the action link "View Attachments". If this link does not appear, there are no attachment files.]

-->Figure Resubmissions:

While revising your submission, we strongly recommend that you use PLOS’s NAAS tool (https://ngplosjournals.pagemajik.ai/artanalysis) to test your figure files. NAAS can convert your figure files to the TIFF file type and meet basic requirements (such as print size, resolution), or provide you with a report on issues that do not meet our requirements and that NAAS cannot fix.

After uploading your figures to PLOS’s NAAS tool - https://ngplosjournals.pagemajik.ai/artanalysis, NAAS will process the files provided and display the results in the "Uploaded Files" section of the page as the processing is complete. If the uploaded figures meet our requirements (or NAAS is able to fix the files to meet our requirements), the figure will be marked as "fixed" above. If NAAS is unable to fix the files, a red "failed" label will appear above. When NAAS has confirmed that the figure files meet our requirements, please download the file via the download option, and include these NAAS processed figure files when submitting your revised manuscript.

# Author comment: Author response to Decision Letter 1

**DOI:** 10.1371/journal.pmen.0000470.r002

16 Apr 2026

Attachment Submitted filename: Responce to Reviewers.docx

# Peer review: Decision Letter 1

**DOI:** 10.1371/journal.pmen.0000470.r003

**Authors:** Karli Montague-Cardoso, Karli Montague-Cardoso

14 May 2026

PMEN-D-25-00452R1

Acceptability of Self-Help Plus and Problem Management Plus interventions by clients and implementation insights from the staff of the Opioid Agonist Treatment programs in Ukraine

PLOS Mental Health

Dear Dr. Klymchuk,

Thank you for submitting your revised manuscript to PLOS Mental Health. After careful consideration of the reviewer reports, we invite you carry out some very minor revisions in line with the remaining reviewer comments. The changes will then be assessed in-house upon resubmission.

Please submit your revised manuscript by Jun 13 2026 11:59PM. If you will need more time than this to complete your revisions, please reply to this message or contact the journal office at mentalhealth@plos.org. When you're ready to submit your revision, log on to https://www.editorialmanager.com/pmen/ and select the 'Submissions Needing Revision' folder to locate your manuscript file.

Please include the following items when submitting your revised manuscript:

- A letter that responds to each point raised by the editor and reviewer(s). You should upload this letter as a separate file labeled 'Response to Reviewers'.
- A marked-up copy of your manuscript that highlights changes made to the original version. You should upload this as a separate file labeled 'Revised Manuscript with Track Changes'.
- An unmarked version of your revised paper without tracked changes. You should upload this as a separate file labeled 'Manuscript'.

Guidelines for resubmitting your figure files are available below the reviewer comments at the end of this letter.

As the corresponding author, your ORCID iD is verified in the submission system and will appear in the published article. PLOS supports the use of ORCID, and we encourage all coauthors to register for an ORCID iD and use it as well. Please encourage your coauthors to verify their ORCID iD within the submission system before final acceptance, as unverified ORCID iDs will not appear in the published article. *Only*  the individual author can complete the verification step; PLOS staff *cannot*  verify ORCID iDs on behalf of authors.

We look forward to receiving your revised manuscript.

Kind regards,

Karli Montague-Cardoso

Staff Editor

PLOS Mental Health

**Journal Requirements:**

If the reviewer comments include a recommendation to cite specific previously published works, please review and evaluate these publications to determine whether they are relevant and should be cited. There is no requirement to cite these works unless the editor has indicated otherwise.

Please review your reference list to ensure that it is complete and correct. If you have cited papers that have been retracted, please include the rationale for doing so in the manuscript text, or remove these references and replace them with relevant current references. Any changes to the reference list should be mentioned in the rebuttal letter that accompanies your revised manuscript. If you need to cite a retracted article, indicate the article’s retracted status in the References list and also include a citation and full reference for the retraction notice.

Additional Editor Comments (if provided):

[Note: HTML markup is below. Please do not edit.]

Reviewers' comments:

Reviewer's Responses to Questions

**Comments to the Author**

1. If the authors have adequately addressed your comments raised in a previous round of review and you feel that this manuscript is now acceptable for publication, you may indicate that here to bypass the “Comments to the Author” section, enter your conflict of interest statement in the “Confidential to Editor” section, and submit your "Accept" recommendation.

Reviewer #1: All comments have been addressed

Reviewer #2: All comments have been addressed

**********

2. Does this manuscript meet PLOS Mental Health’s publication criteria? Is the manuscript technically sound, and do the data support the conclusions? The manuscript must describe methodologically and ethically rigorous research with conclusions that are appropriately drawn based on the data presented.

Reviewer #1: Yes

Reviewer #2: Yes

**********

3. Has the statistical analysis been performed appropriately and rigorously?

Reviewer #1: N/A

Reviewer #2: N/A

**********

4. Have the authors made all data underlying the findings in their manuscript fully available (please refer to the Data Availability Statement at the start of the manuscript PDF file)?

The PLOS Data policy requires authors to make all data underlying the findings described in their manuscript fully available without restriction, with rare exception. The data should be provided as part of the manuscript or its supporting information, or deposited to a public repository. For example, in addition to summary statistics, the data points behind means, medians and variance measures should be available. If there are restrictions on publicly sharing data—e.g. participant privacy or use of data from a third party—those must be specified.

Reviewer #1: Yes

Reviewer #2: No

**********

5. Is the manuscript presented in an intelligible fashion and written in standard English?

PLOS Mental Health does not copyedit accepted manuscripts, so the language in submitted articles must be clear, correct, and unambiguous. Any typographical or grammatical errors should be corrected at revision, so please note any specific errors here.

Reviewer #1: Yes

Reviewer #2: Yes

**********

6. Review Comments to the Author

Please use the space provided to explain your answers to the questions above. You may also include additional comments for the author, including concerns about dual publication, research ethics, or publication ethics. (Please upload your review as an attachment if it exceeds 20,000 characters)

**Reviewer #1:**  Thank you for addressing my comments

**Reviewer #2:**  Overall, the authors have done very well to sufficiently address the comments raised. However, a few issues may need further consideration before publication.

Major comments

• One of the instances of ‘the’ in the title should be removed, i.e., Acceptability of Self-Help Plus and Problem Management Plus interventions among staff and by clients and implementation insights of from the staff of the Opioid Agonist Treatment programs in Ukraine

• Under Materials and Methods, the paragraph starting ‘Phenomenology’ needs one or more references to validate/support the claims being made here.

• The ‘Limitations’ section needs to be before the ‘Conclusions’

Minor comments

• The ‘Sampling strategy’ header should be removed as it is not relevant here.

• Consider presenting the eligibility criteria in sentence rather than dot point format.

• In the discussion, the sentences starting “Again, such results…” and “However, participants…” should be linked together (i.e., as one paragraph) and to the paragraph that precedes it which starts “While the individualized format…”

**********

7. PLOS authors have the option to publish the peer review history of their article (what does this mean?). If published, this will include your full peer review and any attached files.

**Do you want your identity to be public for this peer review?**  If you choose “no”, your identity will remain anonymous but your review may still be made public.

For information about this choice, including consent withdrawal, please see our Privacy Policy.

Reviewer #1: **Yes:** Benjamin ROLLAND

Reviewer #2: **Yes:** Theresa Caruana

**********

[NOTE: If reviewer comments were submitted as an attachment file, they will be attached to this email and accessible via the submission site. Please log into your account, locate the manuscript record, and check for the action link "View Attachments". If this link does not appear, there are no attachment files.]

**Figure Resubmissions:**

While revising your submission, we strongly recommend that you use PLOS’s NAAS tool (https://ngplosjournals.pagemajik.ai/artanalysis) to test your figure files. NAAS can convert your figure files to the TIFF file type and meet basic requirements (such as print size, resolution), or provide you with a report on issues that do not meet our requirements and that NAAS cannot fix.

After uploading your figures to PLOS’s NAAS tool - https://ngplosjournals.pagemajik.ai/artanalysis, NAAS will process the files provided and display the results in the "Uploaded Files" section of the page as the processing is complete. If the uploaded figures meet our requirements (or NAAS is able to fix the files to meet our requirements), the figure will be marked as "fixed" above. If NAAS is unable to fix the files, a red "failed" label will appear above. When NAAS has confirmed that the figure files meet our requirements, please download the file via the download option, and include these NAAS processed figure files when submitting your revised manuscript.

# Author comment: Author response to Decision Letter 2

**DOI:** 10.1371/journal.pmen.0000470.r004

24 May 2026

Attachment Submitted filename: Responce to Reviewers 2.docx

# Editor report: Decision Letter 2

**DOI:** 10.1371/journal.pmen.0000470.r005

**Authors:** Karli Montague-Cardoso, Karli Montague-Cardoso, Karli Montague-Cardoso

29 May 2026

Acceptability of Self-Help Plus and Problem Management Plus interventions among clients and implementation insights from staff of Opioid Agonist Treatment programs in Ukraine

PMEN-D-25-00452R2

Dear Dr. Klymchuk,

We are pleased to inform you that your manuscript 'Acceptability of Self-Help Plus and Problem Management Plus interventions among clients and implementation insights from staff of Opioid Agonist Treatment programs in Ukraine' has been provisionally accepted for publication in PLOS Mental Health.

Before your manuscript can be formally accepted you will need to complete some formatting changes, which you will receive in a follow up email. A member of our team will be in touch with a set of requests.

Please note that your manuscript will not be scheduled for publication until you have made the required changes, so a swift response is appreciated.

IMPORTANT: The editorial review process is now complete. PLOS will only permit corrections to spelling, formatting or significant scientific errors from this point onwards. Requests for major changes, or any which affect the scientific understanding of your work, will cause delays to the publication date of your manuscript.

If your institution or institutions have a press office, please notify them about your upcoming paper to help maximize its impact. If they'll be preparing press materials, please inform our press team as soon as possible -- no later than 48 hours after receiving the formal acceptance. Your manuscript will remain under strict press embargo until 2 pm Eastern Time on the date of publication. For more information, please contact mentalhealth@plos.org.

Thank you again for supporting Open Access publishing; we are looking forward to publishing your work in PLOS Mental Health.

Best regards,

Karli Montague-Cardoso

Staff Editor

PLOS Mental Health

***********************************************************

Reviewer Comments (if any, and for reference):
